# Interventional radiology in European radiology departments: a joint survey from the European Society of Radiology (ESR) and the Cardiovascular and Interventional Radiological Society of Europe (CIRSE)

**DOI:** 10.1186/s13244-019-0698-6

**Published:** 2019-02-13

**Authors:** European Society of Radiology (ESR), Cardiovascular and Interventional Radiological Society of Europe (CIRSE)

**Affiliations:** 1 Am Gestade 1 1010, Vienna, Austria; 2 Neutorgasse 9/6 1010, Vienna, Austria

**Keywords:** Interventional radiology, Workload, Workplace, Department organization

## Abstract

**Objectives:**

To gather information from radiological departments in Europe about the organization and practice of interventional radiology (IR).

**Methods:**

The European Society of Radiology (ESR) and the Cardiovascular and Interventional Radiological Society of Europe (CIRSE) developed an online survey with questions that addressed the organization of IR within radiology departments. The survey was sent to 1180 addresses of department heads throughout Europe.

**Results:**

There were 98 answers (response rate 8.3%) from many European nations, reflecting the situation of IR in Europe.

**Conclusions:**

Five points of action can be suggested based on the survey results. There is a need to assure 24-h service of IR in all radiological departments; networking can be the solution in case staffing problems arise. To attract students, IR needs to be recognized early as a possible career option. Although IR is included in the ESR Curriculum for Undergraduate Radiological Education, this is not the case everywhere, and it must be. There is a “gender issue” in IR since the majority of specialists are male. The lack of role models is probably the main reason why women do not pursue an interventional career. It is, therefore, necessary to increase the number of women in faculty and chair positions to provide a well-balanced leadership team. The field of radiology should work towards recognition of the full clinical role of IR, making efforts to also take into account the “administrative” responsibility throughout the entire process of care for each patient treated by interventional radiologists. Additionally, those radiologists who perform only diagnostic tasks must take an active role in IR. When a situation is encountered which could be amenable to therapy with IR, the radiological report should suggest this form of therapy and the patient should be referred to colleagues in IR.

## Key points

- There is a need to assure 24-h interventional radiology (IR) service in all radiological departments
- To attract more radiologists for IR, it must be recognized early as a career option
- There is an imbalance between male and female interventional radiologists
- IR should be recognized as a clinical part in the entire process of patient care

## Introduction

The European Society of Radiology (ESR) and the Cardiovascular and Interventional Radiological Society of Europe (CIRSE) have developed a combined strategy for joining their forces to address the future of diagnostic (DR) and interventional radiology (IR), with a special focus on the improvement of patient care.

The Board members of the two societies met during both face-to-face and online meetings to discuss this strategy. Among the practical goals set during these meetings, the first and most important one was to gather information about the practice of IR throughout Europe.

It was thought, in fact, that knowing about the current situation of IR would allow to take decisions on where and how to address the efforts of the two societies.

This article reports on the results of a survey which was jointly launched on this topic by the ESR and CIRSE in May 2017. The questions also aimed to evaluate the practice of IR within other, non-radiological, hospital departments in order to understand the relationships among the different performers of these procedures.

## Materials and methods

A questionnaire was developed to acquire data about the practice of IR within radiology departments throughout Europe.

The survey consisted of 30 questions, divided into five groups:

1. Hospital-related: location, dimension, presence, or absence of teaching duties
2. Personal information about the chairperson of the department: type of work (if mainly dedicated to DR, to IR or to both) and if member of ESR, CIRSE, and national radiology and/or interventional radiology societies
3. Related to the medical personnel working in the department: number of radiologists, number of radiologists in training, number of radiologists performing simple interventional procedures (biopsies and drainages), number of interventional radiologists, number of female radiologists, and number of female interventional radiologists
4. Specifically related to IR and organization of the IR services: number of IR procedures/year, types of IR procedures performed (vascular and non-vascular), presence of a dedicated IR unit, of an outpatient service and of case beds available for IR, availability or IR services 24 h/day
5. Dedicated to understanding how much of IR is performed outside radiology, especially neuroradiological and cardiac procedures

The questionnaire was made available online and an invitation to complete it was sent to the approximately 1180 heads of European radiology departments which are contacts of the ESR. The invitation was sent out twice, over a period of 2 months, between April and June 2017.

## Results

Ninety-eight heads of department completed the questionnaire, which equals a response rate of approximately 8.3%. The questionnaire was fully completed by almost all responders; only few of them gave partial answers.

### First group of questions

Answers were received from different parts of Europe; 66.3% came from eight nations (Germany, Turkey, Austria, Croatia, France, Spain, Ireland, and Italy). The distribution according to countries is presented in Table 1.

**Table 1:** Nationality of responders

Germany	15
Turkey	9
Austria	8
Croatia	8
France	8
Spain	7
Ireland	5
Italy	5
UK	4
Bulgaria	4
Switzerland	3
Romania	3
Israel	2
Kazakhstan	2
The Netherlands	2
Sweden	2
Slovenia	2
Denmark	2
Estonia	2
Finland	1
Slovakia	1
Belarus	1
Lithuania	1
Russia	1

There were 51 responses (52.0%) from hospitals with more than 800 beds, 28 (28.6%) from hospitals with 400–799 beds, and 15 (15.3%) from hospitals with 200–399 beds. Only 4 hospitals with less than 199 beds provided an answer. Most answers (94.9%) were from teaching hospitals that employed radiologists-in-training.

### Second group of questions

In regard to the clinical work of the heads of the radiological departments, there are 26 (26.5%) chairpersons who work as diagnostic radiologists; 31 (31.6%) also have some IR duties; 26 (26.5%) work approximately 50% of their time in DR and 50% in IR; 15 (15.3%) work either mostly or exclusively as interventional radiologists. There are 82 (83.7%) who are members of the ESR and 49 (50%) who are members of CIRSE. Among them, 39 are members of both societies; 43 are ESR members only, and 9 are CIRSE members only. In the latter group, there are 7 out of 9 radiologists working only or most of their time in IR and one working 50% DR and 50% IR. Almost all responders (84/98—85.7%) are members of their national radiological society, and 46 out of 98 (46.9%) are members of their national interventional radiology society or of the section on intervention of their national society.

### Third group of questions

The number of radiologists working in radiology departments widely varies, according to the size of the hospital and its needs for imaging services: from less than 10, in 17 small-to-medium-sized hospitals, up to 90, in a very large one. The same can be said for the number of radiologists-in-training in each department: they widely vary, with numbers ranging from 1 to 70. There are two hospitals which have no teaching duties and are without residents and 13 which have more than 21 residents. However, there is a large group of departments (38 out of 98–38.7%) with a number of residents ranging from 6 to 12. Among them, there are both medium-sized (400–799 beds) and large (> 800 beds) hospitals. Except for two medium-sized university hospitals, all hospitals with more than 12 residents in radiology have more than 800 beds.

For the purpose of this survey, an “interventional radiologist” has been defined as a radiologist able to perform “therapeutic interventional procedures” more complex than biopsies and drainages. Based on this definition, there are three small hospitals that do not perform any IR procedures and have no interventional radiologist; most (58 out of 98–62.4%) have a number of interventional radiologists which is 1 to 25% of all radiologists of the department; 25 out of 98 have 26% to 50% of interventional radiologists and only 7 (7.1%) have more than 50%.

The proportion of radiologists who are able to perform simple procedures such as biopsies and drainages is clearly higher. There is not a single department in which such professionality does not exist; there are 30 (32.3%) with 1 to 25% of radiologists able to perform these procedures; 25 (26.9%) with 26 to 50%, 18 (19.4%) with 51 to 75%, and 20 (22.4%) in which more than 75% of radiologists are able to perform biopsies and drainages.

**Fig. 1:**
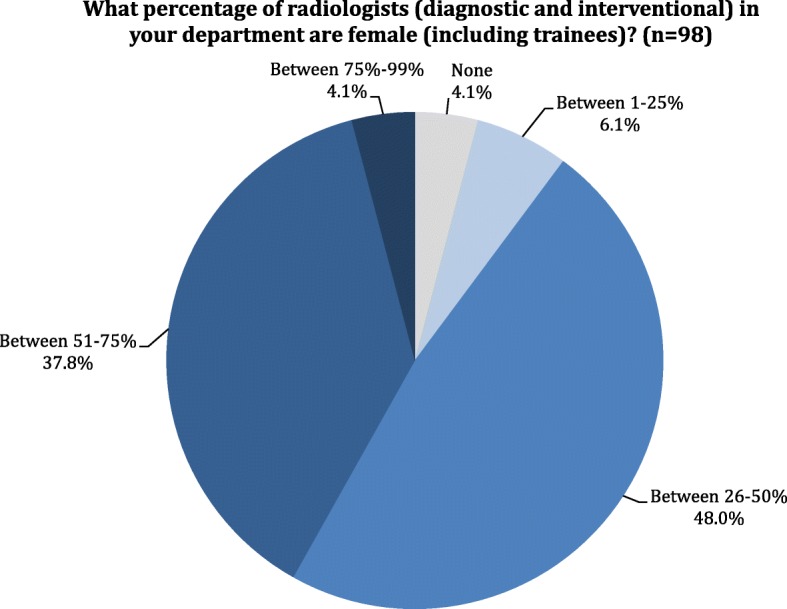
Distribution of the answers to question 15: What percentage of radiologists (diagnostic and interventional) in your department are female (including trainees)?

Gender distribution of radiologists is well-balanced. Only 10 hospitals (10.2%) have no female radiologist or less than 25% of them. There are 47 departments (48%) with 26 to 50% of female radiologists and 37 (37.8%) in which this percentage varies from 51 to 75%. Only four hospitals (4.1%) have more than 75% female radiologists. The percentages of female radiologists in radiology departments are presented in Fig. 1.

**Fig. 2:**
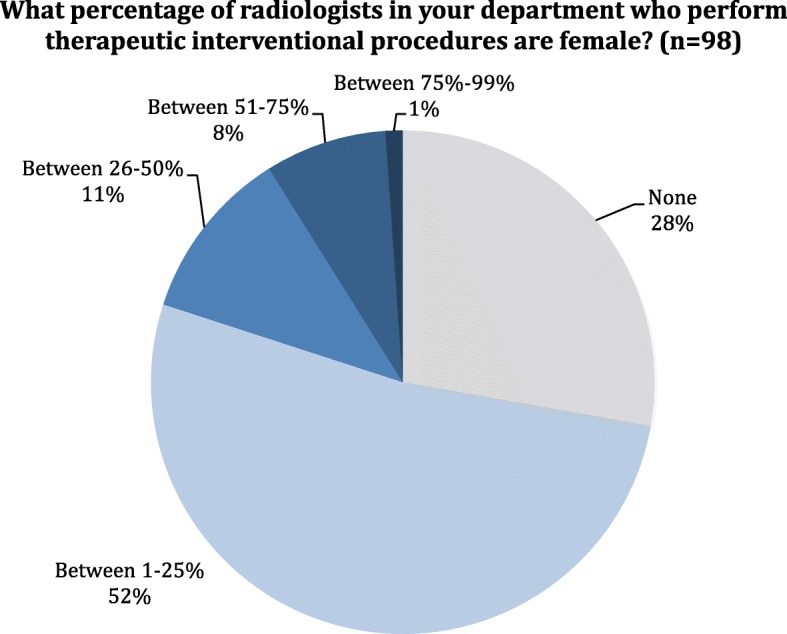
Distribution of the answers to question 16: What percentage of radiologists in your department who perform therapeutic interventional procedures are female?

On the contrary, the majority of interventional radiologists are male. There are 25 departments (27.8%) in which interventional radiologists are only males and 47 (52.2%) in which females only represent 1 to 25% of all interventional specialists. The percentages of female interventional radiologists are presented in Fig. 2.

### Fourth group of questions

The fourth group of questions was directly related to the organization of IR and the volume and types of procedures performed. More than 500 procedures/year are performed in 68 out of 98 departments (69.4%), and between 201 and 500 procedures/year are performed in 15 hospitals (15.3%). Fewer procedures (between 1 and 200) are performed in only 13 hospitals (13%). Two departments (small ones, without IR specialists) do not perform image-guided interventions.

An “interventional unit” under a specific leader is present in 64 out of 98 cases (65.30%), with interventional radiologists running an outpatient service in 57 hospitals (58.8%). The latter is located within the radiology department in 34 cases (60.7% of the 56 chairpersons who provided response to this question). However, day-case beds are available for radiologic-guided interventions in 30 cases only (30.9%). The question on how these beds are organized was answered by 28 responders: they are run in cooperation with another clinical team in 12 cases (42.8%), run by another clinical team in 1, and run by radiologists or IR specialists in only 15 (53.6%) cases. In fact, the percentage of patients referred for IR procedures who are directly admitted under IR widely varies. In only 33 departments (34%), they are all (or most of them are—from 75 to 99%) admitted under IR; in 44 cases (44.3%), they are all (or the large majority—up to 75%) admitted under other wards or departments. Ward rounds are performed by IR specialists in 55 out of 97 departments (56.7%).

The IR service is arranged to cover emergencies on a 24/7-basis in 60 out of 97 hospitals.

**Fig. 3:**
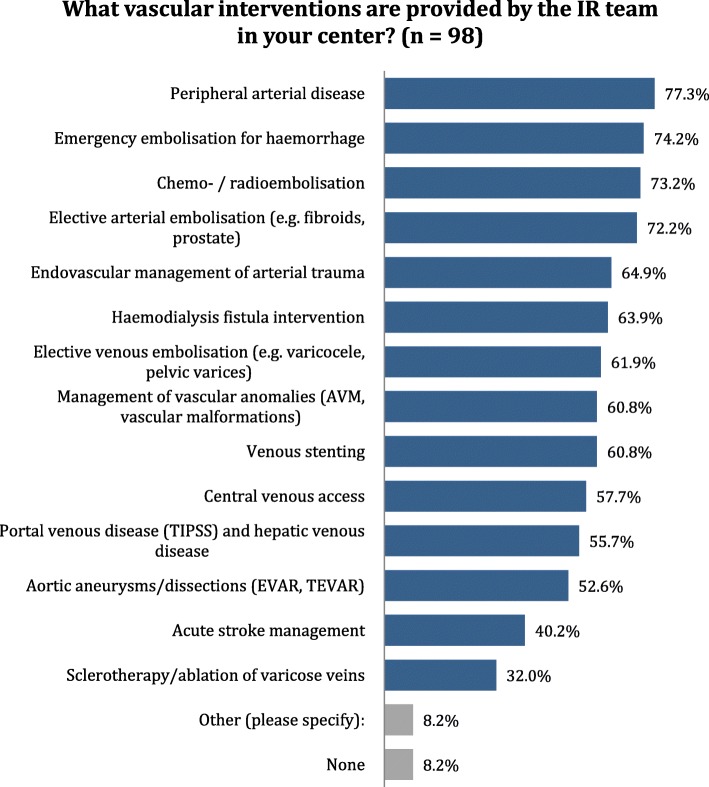
Distribution of answers to question 27: What vascular interventions are provided by the IR team at your center?

**Fig. 4:**
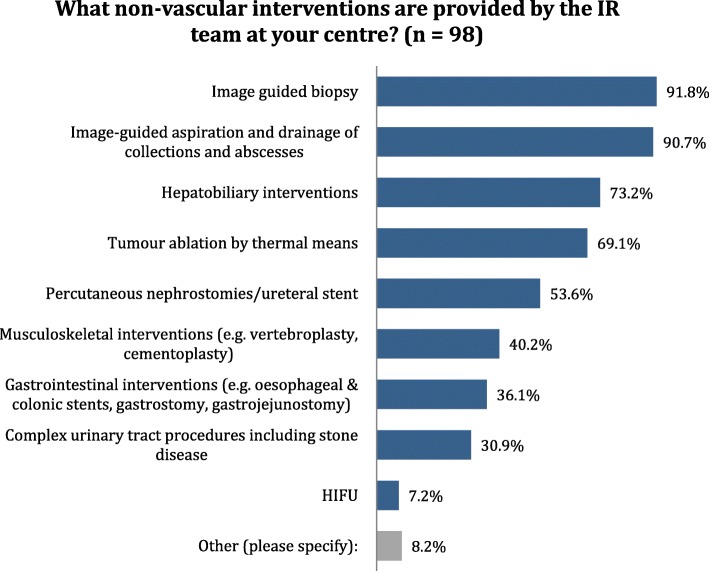
Distribution of answers to question 28: What non-vascular interventions are provided by the IR team at your center?

A large variety of vascular and non-vascular interventional procedures are performed (Figs. 3 and 4).

About three quarters (71 out of 97–73.2%) of interventional radiologists perform interventional procedures in all fields, while in 26 hospitals (26.8%), they have specialized based on the system/organ they most frequently treat. This usually happens in centers with a high number of yearly procedures. In most of these centers, interventional specialists dedicate themselves to one clinical area: to neurointerventions, to vascular procedures, or to non-vascular treatments (usually interventional oncology). Furthermore, there are specialists who are specifically dedicated to musculoskeletal interventions.

### Fifth group of questions

Neurointerventional procedures are performed in 72 centers, mostly (45 out of 72–62.2%) by interventional neuroradiologists. Interventional radiologists are directly involved in them in 19 out of 72 (26.4%) departments. There are, however, 8 hospitals in which neurointerventional services are provided by other specialists, such as neurosurgeons, cardiologists, traumatologists, or neurologists.

Cardiac interventional procedures are performed in 84 hospitals. The service is provided by interventional radiologists in only 1 of them. In all others, it is run by cardiologists or cardiac surgeons.

## Discussion

Several considerations can be drawn from the results of this study.

The first one relates to the response rate (8.3%) to the survey itself. This figure is in line with the response rates to this type of survey, although, admittedly, it is relatively low. The many commitments of department head could explain the lack of more answers, rather than a low interest for the activities of the two societies that launched the survey.

Answers to the first group of questions show that these came from many different European nations, indicating that the responses can be considered as representative of the situation of IR throughout Europe. Furthermore, most responses came from large hospitals with teaching duties, suggesting that the data can be considered as a good reflection of IR practice at the institutions where residents are trained.

When it comes to the second group of questions, it must be noted that almost half of the heads of departments who responded to the questionnaire (41 out of 98–41.83%) are directly involved (either for most of their clinical work or half of it) in IR activities. This, in our opinion, suggests that interventional radiologists are an integral part of radiology departments and are involved in organizational issues of both the diagnostic and interventional parts of our work. However, given the topic of the questionnaire, it might also have been that the department heads with special interest in IR felt more interested in the survey; then, the percentage of IR responders could be higher than the actual percentage of IR specialists leading radiology departments. The criteria on which each department head has decided to join as a member of one of the two societies, or both, are less clear but are probably related to the specific scientific interests of each of them.

The number of radiologists in each department widely varies but is, however, correlated to the dimensions of the hospitals and their imaging needs. The same can be said about the number of trainees. In regard to interventional activities, simple interventional procedures, such as image-guided biopsies and drainages, can be performed in all departments. There are only two small hospitals that have no specialists with IR expertise and do not perform more complex procedures. One of them has teaching duties, and there are three additional hospitals with only minimal interventional activity (between 1 and 50 procedures/year) in which there are radiology trainees. It is hoped that the exposure to interventional procedures is provided to residents frequently at other sites of a complete training network.

There are clear-cut differences in the gender distribution of diagnostic vs interventional radiologists. Women account for about half (and in some hospitals even more) of all radiology specialists; on the other hand, they are a small minority among those dedicated to IR. It seems that IR is not attractive to women or that women feel discouraged to pursue a career in this field. Radiation protection considerations, the need for high flexibility for covering on-calls and heavy workload, with the consequent difficulties in balancing roles at home and work, are possible factors that explain this low number. These factors have been evaluated in regard to females who work in diagnostic radiology, but the real reasons why few women choose IR are poorly understood [1]. As a matter of fact, only radiation protection considerations and a lack of opportunity have been suggested as the reasons why women do not choose an interventional career in the similar field of interventional cardiology [2].

Most departments (83 out of 98–84.69%) have an IR workload higher than 201 procedures/year, and 68 out of 98 (69.38%) perform more than 500 interventions. This means that, in the majority of cases, the clinical needs are met and, even more important, that there is enough IR case-load to guarantee expertise maintenance and training of the future generations of interventional radiologists. Furthermore, a large variety of procedure types is performed in most departments; again, an essential requirement to provide both service in all aspects of IR and complete training.

An “interventional radiology unit” under a specific leader is present in 64 out of 98 cases (65.30%); interventional radiologists run an outpatient service in 30 hospitals, and this is located in the radiology department in 34 cases (the majority of responders to this question). However, there is still a long way to go before getting recognition of the role of IR within the hospital organization. Availability of case-beds for pre- and post-procedural care of patients undergoing interventional procedures is somewhat limited (30.9%). Furthermore, they are run by radiologists in slightly more than half of the cases. The same can be said of the percentage of patients referred for interventional procedures who are admitted under IR; this happens in about one third of cases only. There are a number of factors causing this lack of recognition; among them, the willingness of clinical specialists to keep control on patients’ admission to the hospital wards and on referrals from outside doctors are probably among the most important. However, if we consider that IR radiologists perform ward rounds to check their patients in 56.7% of cases only, and that the IR service is arranged to cover emergencies on a 24/7-basis in only 60/97 centers (60.8%), there is some responsibility on our side too. Staffing and financial issues may make the organization of a 24/7 on-call IR service difficult to organize. Nevertheless, the role of IR in emergencies is of great importance, can be life-saving, and underlines the importance of radiology in patients’ care. Then, there are both clinical and “political” reasons to make all efforts to provide this service. Furthermore, following the patients’ status after intervention is a way to affirm our role of radiologists as clinicians, and it is, therefore, necessary to take full responsibility for the results of interventional procedures, to actively care for the patient until his/her final recovery and discharge, and to “show” it.

About three quarters of interventional radiologists perform interventional procedures in all fields, while in 26 hospitals they have specialized according to the system/organ they treat most frequently. This usually happens in centers with high numbers of procedures and in which there is a relatively high number of radiologists dedicated to IR. These data underline, on one side, the tendency of interventional radiologists to apply their knowledge and skills to all fields of the subspecialty. On the other side, however, they demonstrate that the patterns of practice may change according to the clinical needs, the workload, the number of specialists available, and the sizes of the hospital. On the other hand, presence of musculoskeletal radiologists who perform interventional procedures in their field may also indicate that diagnostic radiologists with special interest in one subspecialty can develop interventional capabilities in that field and start treating patients.

The relationship between radiologists and clinical specialists is not always easy. Turf battles exist both in diagnostic and in interventional radiology. Cardiac interventions are performed by cardiologists, angiologists, or cardiac surgeons almost everywhere; unfortunately, this can be considered as a “lost” field. The situation is different when it comes to neurointerventions. These are still in radiological hands. Interventional neuroradiologists and interventional radiologists, in fact, perform them in the majority of cases (65.97%). However, there are neurologists, neurosurgeons, traumatologists, and even cardiologists who perform such interventions in some centers.

## Action points

Many surgical procedures have been replaced or enhanced by the provision of IR services, and IR procedures have enabled new, not previously, feasible treatments for patients. Furthermore, an IR approach is less invasive to the patient, reducing morbidity and mortality, and allows for more rapid recovery.

Interventional radiologists are radiologists who have undergone additional specialist training in the practical elements of IR. For the purposes of this survey, we have defined IR as those radiologists who can perform “therapeutic procedures” more complex than just biopsy and drainages.

After analysis of this survey, the two societies would like to underline a few specific points aimed at improving the availability of these services.

Access to robust 24-h IR coverage should be a priority for all acute care hospitals. For this duty, the number of interventional radiologists must be sufficient. However, with many hospitals having limited or, in some instances, no direct access to IR services, the provision of IR services remains variable. Our data demonstrate that the situation in Europe is somewhat better than that shown in a recent survey performed in the UK, where less than one third of hospitals are able to provide comprehensive out-of-hours IR care, potentially putting many patients at risk [3]. In Europe, about 60% of radiological departments can cover IR emergencies. This is not enough, however, and is a problem that needs to be urgently addressed. Services consisting of six or more interventional radiologists will usually be able to provide an effective and sustainable service. If this is not the case, the first solution should be networking among hospitals. This can be difficult to organize, especially if there is competition among different hospitals or radiological units, but would allow providing emergency services on a 24-h basis within a region and even to provide separate vascular and non-vascular rota in order to give highly specialized services.

The main cause of the problem mentioned above is a significant shortage of interventional radiologists. Both societies believe that this problem may lead to a loss of ground of IR towards other medical specialties and make the specialty of radiology, as a whole, more fragile.

IR needs to be recognized as a career early, during the years of medical school, to be able to attract students.

In a survey of medical students performed in the USA, respondents were asked to rate their diagnostic and interventional radiology exposure during medical school. The majority of the respondents described their interventional radiology exposure as minimal or none, in contrast to diagnostic radiology exposure, which was graded as average or above [4]. It has been shown that exposure to this subspecialty of radiology makes medical students keen to learn more about it and, possibly, may help to recruit new talents [5]. Both societies agree that the whole spectrum of radiology must be exposed to medical students, and, therefore, interventional radiology has to be included. This is underlined in the “Curriculum for Undergraduate Radiological Education” of the ESR (developed with contribution of CIRSE) [6] where all the different aspects of radiology are duly considered and have to be put into practice everywhere in Europe.

Our survey clearly shows a gender imbalance in interventional radiology since the majority of interventional radiologists are male. IR, in fact, remains a male-dominated specialty in comparison to other subspecialties of radiology. Although more and more women are entering careers in medicine—46% of all physicians in training and almost half of all medical students are women—according to an analysis of the Association of American Medical Colleges (AAMC) [7], there are some specialties which are rarely chosen by female physicians, such as orthopedic surgery, urology, and interventional specialties as a whole [1, 2]. It is not clear which factors are behind this phenomenon. There are data suggesting that radiation exposure is the main reason why female medical students do not pursue interventional radiology, and interestingly, this reason is mirrored in their male counterparts. When comparing deterring factors between female and male respondents, however, the two significant gender differences were call responsibilities and male predominance [1]. Lack of opportunity for female applications has been suggested as another important reason [2]. This is a problem that is linked to the present-day male predominance and is a difficult one with which to cope. The paucity of role models is probably the main reason why women do not pursue an interventional career. It is, therefore, essential to increase the number of women in IR faculty and chair positions to provide a diverse, well-balanced leadership team [8]. Addressing this issue will improve the diversity in our candidates’ pool. It is a matter that is not only important for us: diversity, in fact, is thought to improve patient communication and decrease health disparity [1].

Additional attention should be given to the clinical implications of IR. Interventional radiologists play a clinical and therapeutic role that is essential for the “visibility” of our discipline. They examine the patient, find the indications to the procedure, obtain consent, and do both the procedure and its follow-up. Then, they have full pre-, peri-, and post-procedural responsibility for the patient [9, 10]. Many of these procedures can be performed on a day-case basis, and IR offers an opportunity to deliver cost-effective care when day-case and outpatient facilities are used appropriately [3].

This should be recognized, by the patients themselves and within the hospital organization, but this is not always the case. Day-case beds are allocated to radiology in less than 50% of departments, and often only in cooperation with clinical teams. Radiology as a whole should work to obtain recognition of the full clinical role of IR, making all efforts to also take “administrative” responsibility for the full episode of care of each patient treated by interventional radiologists.

Working together, from a department perspective, means that those radiologists who perform only diagnostic tasks must also take an active role in IR. Adding recommendations on further studies or therapeutic procedures in the radiologic report is a relatively common event. When a situation that recommends therapy with IR is encountered, the radiological report should suggest it, and the patient should be referred to IR colleagues. The same conduct should also be taken during clinical-radiological meetings if an interventional radiologist is not present. This could increase the number of referrals to IR and, most probably, of appropriate ones. Indications for interventional procedures should be well-known by all radiologists. Sharing knowledge of IR among our colleagues in diagnostic radiology and putting it into practice will surely enhance our role as physicians and will certainly provide a better service for our patients.
